# PI3 K/Akt/mTOR-mediated translational control regulates proliferation and differentiation of lineage-restricted RoSH stem cell lines

**DOI:** 10.1186/1750-2187-2-9

**Published:** 2007-09-25

**Authors:** Jianwen Que, Qizhou Lian, Reida M El Oakley, Bing Lim, Sai-Kiang Lim

**Affiliations:** 1 Dept. of Surgery, National University of Singapore, Lower Kent Ridge Road, Singapore 117597; 2 Genome Institute of Singapore, 60 Biopolis Street, Singapore 138672; 3 Beth Israel Deaconess Medical Center, Harvard Medical School, 4 Blackfan Circle, Boston, MA, USA 02115; 4 Institute of Medical Biology, 11 Biopolis Street, Helios #02-02, Singapore 13866

## Abstract

**Background:**

We have previously derived highly similar lineage-restricted stem cell lines, RoSH and E-RoSH cell lines from mouse embryos and CD9^hi ^SSEA-1^- ^differentiated mouse embryonic stem cells, respectively. These cell lines are not pluripotent and differentiate readily into endothelial cells *in vitro *and *in vivo*.

**Results:**

We investigated the signaling pathway that maintains proliferation of these cells in an undifferentiated state, and demonstrate that PI3 K/Akt/mTOR, but not Raf/MEK/Erk, signaling in these cells was active during proliferation and was downregulated during endothelial differentiation. Inhibition of PI3 K/Akt/mTOR signaling, but not Raf/MEK/Erk, reduced proliferation and induced expression of endothelial specific proteins. During differentiation or inhibition of PI3 K/Akt/mTOR signaling, cyclinD2 transcript abundance in ribosome-enriched RNA but not in total RNA was reduced with a corresponding reduction in protein level. In contrast, transcript abundance of endothelial-specific genes e.g. *Kdr*, *Tek *and *Pdgfrα *in ribosome-enriched RNA fraction was not reduced and their protein levels were increased. Together these observations suggested that translational control mediated by PI3K/Akt/mTOR signaling was critical in regulating proliferation and endothelial differentiation of lineage-restricted RoSH-like stem cell lines.

**Conclusion:**

This study highlights translation regulation as a critical regulatory mechanism during proliferation and differentiation in stem cells.

## Background

Embryonic stem cells (ESCs) are pluripotent stem cells capable of differentiating into cells of all three germ layers, making ESCs an ideal source of cells for regenerative therapy for many diseases and tissue injuries [1,2]. However, this property of ESCs poses a unique challenge of having to generate therapeutically efficacious quantity of appropriate cell types without being contaminated by potentially deleterious cell types. Recently, we have generated lineage-restricted stem cell lines with endothelial potential termed RoSH and E-RoSH lines from mouse embryos and mouse ESCs (mESCs), respectively [3,4]. RoSH and E-RoSH lines are derived from CD9^hi^, SSEA-1^- ^cells in embryo and ESC-derived embryoid body cultures. Despite their different tissue source of origin, both RoSH and E-RoSH cell lines are highly similar with an almost identical gene expression profile [3,4]. They differentiate efficiently into endothelial cells when plated on matrigel or when transplanted into appropriate animal models. They can be propagated in culture as cell lines and have a population doubling time of 12–15 hours. They are also highly amendable to subcloning from single cells. As therapeutic agents, such cell lines will have several distinct advantages over pluripotent ESCs. One, they are not pluripotent and therefore, cannot form teratoma. Two, these lines as a consequence of their reduced potency differentiate efficiently into their target cells, specifically endothelial cells and formed vascular structures *in vitro *and *in vivo*. Third, as clonable cell lines, these cells can be produced as highly homogenous cell population on a large scale.

To better understand and manipulate proliferation and differentiation of these cell lines, we here investigated two major signaling pathways known to regulate cellular proliferation, PI3 K/Akt/mTOR [5] and Raf/MEK/Erk [6] in a representative RoSH/E-RoSH cell line, RoSH2 cell line. We found that PI3 K/Akt/mTOR but not Raf/MEK/Erk signaling was downregulated during differentiation. Inhibitors of PI3 K/Akt/mTOR but not Raf/MEK/Erk signaling reduced proliferation of RoSH cells and induced expression of endothelial specific proteins. Downregulation of PI3 K/Akt/mTOR signaling during differentiation or treatment with rapamycin, a specific inhibitor of mTOR was associated with downregulation of mTOR-mediated translational control. Ribosomal recruitment of 5' tract of pyrimidines (TOP)-containing RNAs e.g. ribosomal protein L5 RNA transcripts was reduced. Transcript abundance of cyclinD2, a G1 cyclin that promotes G1/S progression was also reduced in ribosome-enriched RNA fraction but not total RNA, accompanied by a corresponding decrease in cyclin D2 protein level. In contrast, transcript abundance of endothelial specific proteins, Kdr (or Flk1), Tek (or Tie2) and Pdgfrα was increased in ribosome-enriched RNA fraction and protein levels were increased.

## Results

### Proliferation rate of self-renewing RoSH2 cells was reduced when induced to differentiate

**Figure 1:**
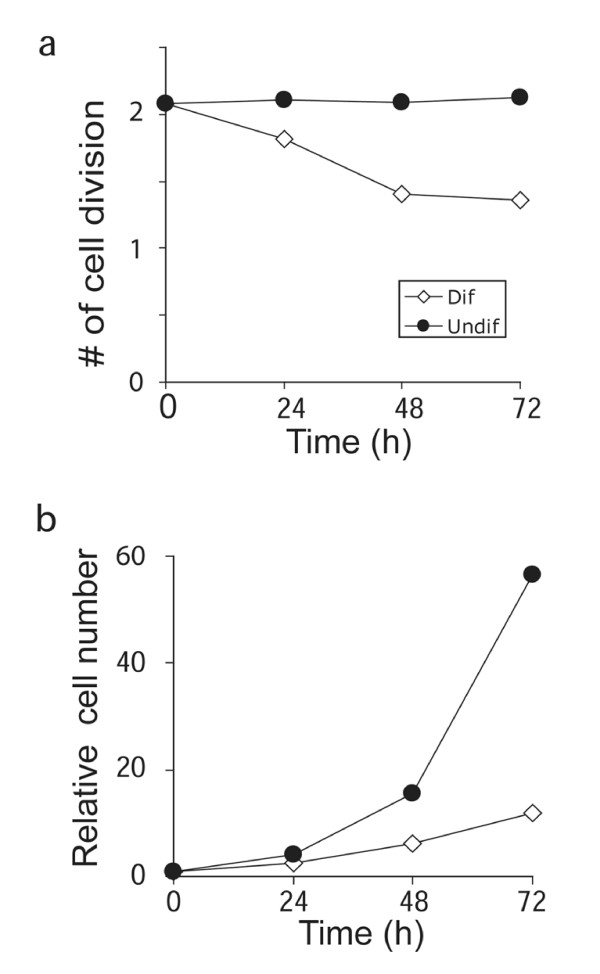
**Growth and cell cycle regulation during endothelial differentiation**. a) Rate of cell proliferation. RoSH2 cells were labeled with CFDA, a cell-permeable fluorescent dye, and then cultured for 24 hours. They were then plated on gelatin-coated plates under non-differentiating condition or on matrigel-coated plate under differentiating condition. At 0, 24, 48 and 72 after plating, cells were harvested, median cellular fluorescence was measured by flow cytometry and the number of cell divisions was calculated as a function of the loss in fluorescence for every twenty-four hour; b) the cell numbers were counted and normalized against cell count at time 0. Cell number at time 0 was designated as one.

To determine if proliferation in RoSH-like cell was reduced during differentiation, a representative embryo-derived clonal RoSH line, RoSH2 cell line that is highly similar to ESC-derived RoSH, E-RoSH cell lines was used for this study [3,4]. The rate of cell division before and after induction of differentiation was determined by pre-labeling cells with cell-permeable CFDA fluorescent dye [7] and the cells were then plated on either gelatin or matrigel. The rate of cell division was calculated as a function of the loss in cellular fluorescence during a 24 hour period (see method). Undifferentiated cells plated on gelatin maintained a constant rate of cell division from 2.08 ± 0.02 to 2.13 ± 0.03 (n = 3) over a 72 hour period while the rate of cell division in differentiating cells plated on matrigel was significantly reduced from 2.08 ± 0.04 (n = 3) to 1.4 ± 0.05 (n = 3) divisions per 24 hours (p < 0.05) (fig 1a). The reduced rate of cell division after induction of differentiation was reflected in a 4-fold reduction in cell population in the differentiating cell culture at 72 hours (fig 1b).

### PI3K/Akt and not Raf/MEK/Erk regulates proliferation of RoSH2 cells

**Figure 2:**
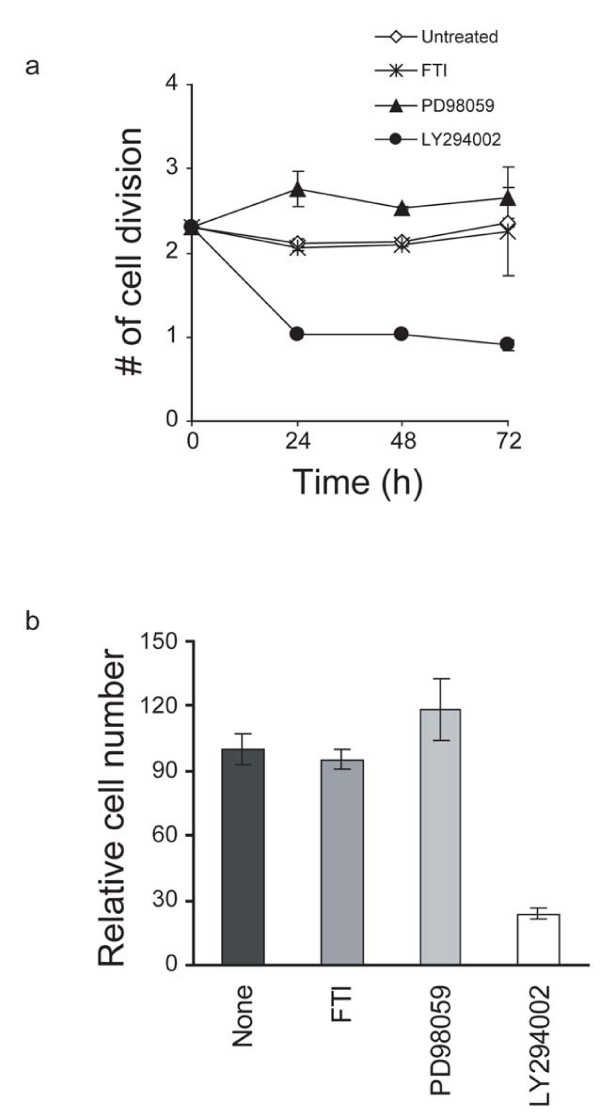
**Effects of inhibiting PI3 K/PI3K/Akt or Raf/MEK/Erk on growth of RoSH2 cells**. RoSH2 cells were pre-labeled with CFDA, cultured on gelatin-coated plates for 24 hours. One quarter of the plates were not treated with any inhibitor (N), one quarter with 10 μM Ras inhibitor, FTase inhibitor III (FTI), one quarter with 50 μM MEK inhibitor PD98059 (PD), and the last quarter with LY 294002 (LY). Each of the media with the appropriate inhibitors was replaced every twenty-four hours. At 0, 24, 48 and 72 hours after plating, cells were harvested and were assayed for cellular fluorescence by flow cytometry. The number of cell cycles for every 24 hours was calculated as described in Materials and Methods. b) Effects of FTI and PD98059 on cell numbers. Twenty-four hours after treatment with FTI, PD98059 or a combination of both drugs, the cell numbers were counted and normalized against that of untreated cells. Cell number at time 0 was designated as one.

To determine if PI3K/Akt and/or Raf/MEK/Erk signaling regulate proliferation in self-renewing RoSH2 cells, the cells were treated with LY294002 (a specific inhibitor of PI3K), FTI (an inhibitor of Raf) and PD98059 (an inhibitor of MEK) and the rate of cell division was measured. At 72 hours after treatment, the rate of cell division in untreated, FTI and PD98059 treated RoSH2 cells were not significantly different at 2.36 ± 0.09 (n = 3) 2.25 ± 0.12 (n = 3) and 2.48 ± 0.16 (n = 3), respectively (fig 2a). However, the rate of cell division was significantly reduced to 0.90 ± 0.07 (n = 3) in LY294002 treated RoSH2 cells (p < 0.01) (fig 2a). 72 hours after drug treatment, cell population size in FTI and PD98059 treated RoSH2 cell cultures were comparable to that of untreated cells. LY294002 treated RoSH2 cell population was much reduced and was 23.9 ± 2.89% (n = 3) of untreated cultures. These observations suggested that PI3 K/Akt signaling and not Raf/MEK/Erk is the major signaling pathway regulating proliferation in RoSH2 cells.

### PI3 K/Akt mediates proliferation through a rapamycin-sensitive pathway

**Figure 3:**
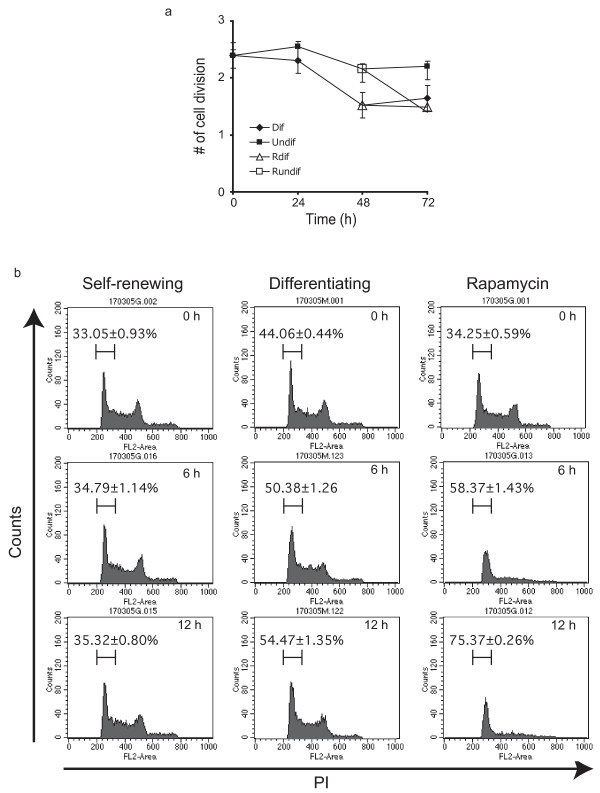
**Growth and cell cycle regulation during endothelial differentiation and rapamycin treatment**. a) Rate of cell division. RoSH2 cells were labeled with CFDA, a cell-permeant fluorescent dye, cultured for 24 hours and re-plated on gelatin-coated plates to be maintained as undifferentiated cells (Undif) or on matrigel to induce differentiation (Dif). Cells were harvested at 0, 24, 48 and 72 hours. At 48 hours after replating, half of the remaining plates of cells under undifferentiating condition or differentiating condition were treated with 50 ηM rapamycin (R undif and Rdif, respectively). Median cellular fluorescence of the harvested cells was measured by flow cytometry and the number of cell divisions was calculated as a function of the loss in fluorescence; b) Cell cycle progression during endothelial differentiation. RoSH2 cells were plated on either gelatin-coated plate (self-renewing) or matrigel (differentiating) and labeled with BrdU for 16 hours. After removing BrdU, half of the gelatin-coated plates were treated with 50 ηM rapamycin. At 0, 6 and 12 hours, cells were harvested, stained with anti-BrdU and PI. DNA content of BrdU-labeled cells as measured by PI was analyzed by flow cytometry.

We next determined if PI3 K/Akt regulates proliferation through its downstream target, the mammalian target of rapamycin (mTOR), a highly conserved serine/threonine kinase and a major regulator of cell growth and proliferation [5]. Rapamycin is a specific inhibitor of mTOR [8]. At 50 nm, rapamycin significantly reduced the rate of cell division in undifferentiating cells from 2.2 ± 0.09 (n = 4) to 1.4 ± 0.04 (n = 4) (p < 0.05) in 24 hours (fig 3a). This reduced rate was greater than the rate of 0.90 ± 0.07 (n = 3) elicited in LY294002 treated RoSH2 cells, suggesting that a rapamycin-sensitive pathway partially mediates PI3 K regulation of cell division in undifferentiated RoSH cells. Rapamycin treatment had no significant effect on the rate of cell division in differentiating RoSH cell cultures. 24 hours after treatment, the rate of cell division was remained unchanged from 1.5 ± 0.22 (n = 4) to 1.5 ± 0.12 (n = 4) (fig 3a). Increasing the concentration of rapamycin from 50 to 200 ηM had no significant effect on the rate of cell division (data not shown). Therefore, differentiation-associated decrease in cell division was mediated by downregulation of a rapamycin-sensitive pathway, possibly mTOR signaling such that further inhibition by rapamycin elicited no further decrease in the rate of cell division. As inhibition of mTOR signaling is known to inhibit cell cycle through reduced G1/S progression [9,10], dividing RoSH2 cells were labeled with BrdU and the distribution of BrdU-labeled cells in different phases of cell cycle was determined by DNA content. Undifferentiated self-renewing cells maintained 34–35% of cells in G1 phase (fig 3b). Rapamycin and differentiation induced accumulation of cells in G1 phase. In 12 hours, there was an increase from 34.25 ± 0.59% to 75.37 ± 0.26% (n = 3; p < 0.05) in rapamycin-treated cells (fig 3b) and a slower increase from 44.06 ± 0.44% to 54.47 ± 1.35% (n = 3; p < 0.05) in differentiating RoSH2 cells. The slower increase in differentiating RoSH2 cells was not unexpected as the inhibition of mTOR during differentiation unlike rapamycin-induced inhibition will have to be effected indirectly through a cascade of cellular events. Therefore rapamycin treatment and induction of differentiation elicited similar cellular responses i.e. reduced cellular proliferation and accumulation of dividing cells in G1 phase suggesting that rapamycin-sensitive PI3K/Akt/mTOR is a major signaling pathway in promoting proliferation of undifferentiated RoSH2 cells and inhibiting differentiation.

### Downstream effectors of PI3 K/Akt/mTOR signaling, Rps6kb1 (or S6K1) and Eif4ebp1 (or 4EBP1) were dephosphorylated during differentiation of RoSH2 cells

**Figure 4:**
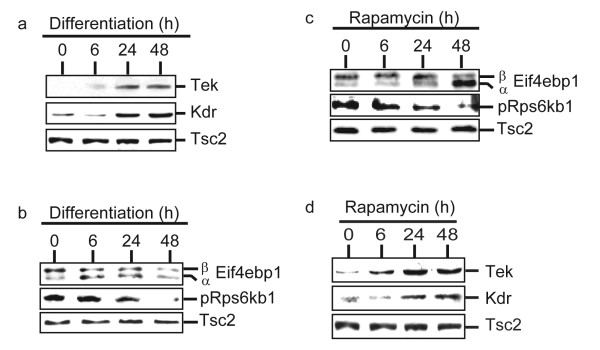
**Phosphorylation of mTOR targets and expression of endothelial markers during differentiation and rapamycin treatment**. At 0, 6, 24 and 48 hours after RoSH2 cells were induced to differentiate by plating on matrigel-coated plates or treated with 50 ηM rapamycin, the cells were harvested for western blot analysis and probed for a, d) endothelial markers, Tie-2 and Flk-1; b, c) Eif4ebp1 and phosphorylated Rps6kb1. Tsc2 protein was used as an internal control for loading between lanes and between blots.

To investigate the molecular mechanism of PI3 K/Akt/mTOR in mediating proliferation, we determined the phosphorylation status of the major downstream effector molecules of PI3 K/Akt/mTOR signaling, eIF4E-binding protein 1, Eif4ebp1 (previously known as PHAS-I and mouse homolog of human 4EBP1) and the ribosomal protein S6 kinase 1, Rps6kb1 (previously known as p70s6k or S6K1) [5]. It is well established that rapamycin-sensitive mTOR (or Frap1) activity regulate G1/S phase progression in cell cycle through Rps6kb1 and Eif4ebp1 [9,11]. During differentiation of RoSH2 cells when the level of endothelial specific markers such as Tek and Kdr (fig 4a) were increasing, we observed a corresponding decrease in phosphorylated Rps6kb1 and the ratio of hyperphosphorylated β to hypophoshorylated α isoform of Eif4ebp1 decreased (fig 4b). Consistent with this, treatment of RoSH2 cells with rapamycin also inhibited phosphorylation of Rps6kb1 and Eif4ebp1 (fig 4c) and unexpectedly increased expression of endothelial specific proteins such as Tek and Kdr (fig 4d).

### Phosphorylation of Rps6kb1 and Eif4ebp1 is mediated by PI3 K/Akt/mTOR and not Raf/MEK/Erk signaling

**Figure 5:**
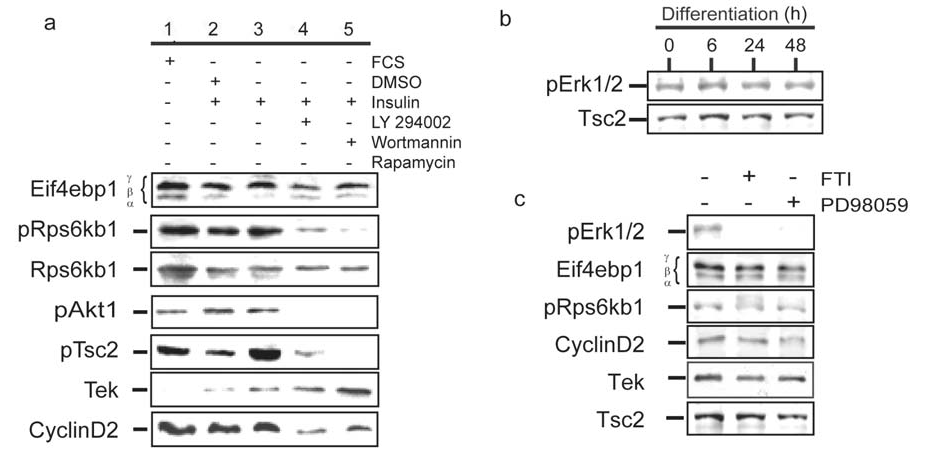
**PI3K and Raf/MEK/Erk signaling in RoSH2 cells**. a) Effects of PI3 K/Akt inhibitors. Undifferentiated RoSH2 cells were plated and cultured for 24 hours, then serum-starved for 20 hours before culturing in media with 50 μM LY294002 dissolved in DMSO, 100 nM wortmannin or 0.1% (v/v) DMSO for 15 min and then challenged with 100 nM insulin for 20 minutes. Cell lysates were prepared and assayed by western blot analysis; b) Raf/MEK/Erk signaling during differentiation. At 0, 6, 24 and 48 hours after plating RoSH2 cells on matrigel, cell lysates were prepared and analyzed by western blot assay for phosphorylated ERK1/2.. c) Effects of inhibiting Raf/MEK/Erk signalingRoSH2 cells were treated with 10 μM or 50 μM Ras inhibitor FTase inhibitor III, (FTI) and MEK inhibitor PD98059 for three hours and cell lysates were analyzed by western blot assays.

To confirm that PI3K/Akt signaling in RoSH2 cells was transduced through mTOR, RoSH2 cells were serum-starved and then challenged with insulin in the presence of LY294002 and wortmannin. Both wortmannin and LY294002 attenuated insulin-induced phosphorylation of Akt and reduced phosphorylated Tsc2 levels (lane 4, 5; fig 5a) relative to that in undifferentiated RoSH2 cells (lane 1; fig 5a), in serum starved RoSH2 cells treated with DMSO and insulin (lane 2; fig 5a) or in serum starved RoSH2 cells treated with insulin (lane 3; fig 5a). As unphosphorylated Tsc2 is known to inhibit mTOR [12], our observed reduction in phosphorylated Tsc2 will result in increased inhibition of mTOR. This was confirmed by the reduced ratio of the highly phosphorylated γ and β isoforms to the less phosphorylated α isoform of Eif4ebp1 and reduced level of phospho-Rps6kb1 (lane 4, 5; fig 5a) relative to that in undifferentiated RoSH2 cells (lane 1; fig 5a), in serum starved RoSH2 cells treated with DMSO and insulin (lane 2; fig 5a) or in serum starved RoSH2 cells treated with insulin (lane 3; fig 5a). In all treatments, the level of Rps6kb1 was constant.

As the classical MAP kinase (Erk) pathway is sometime implicated in the regulation of mTOR and downstream effectors, Rps6kb1 and Eif4ebp1 [13-21], the level of pERK1/2 was determined and found to be constant during differentiation (fig 5b). Inhibition of Raf/MEK/Erk pathway by treating cells with farnesyl transferase inhibitor (FTI), a Ras inhibitor, or PD98059, a MEK inhibitor abolished phosphorylation of pERK1/2 without significant effects on phosphorylation of Eif4ebp1 or Rps6kb1. These observations suggest that mTOR signaling in RoSH2 cells is downstream of PI3 K/Akt and not Raf/MEK/Erk pathway.

### Rapamycin-sensitive translational regulation during differentiation

**Figure 6:**
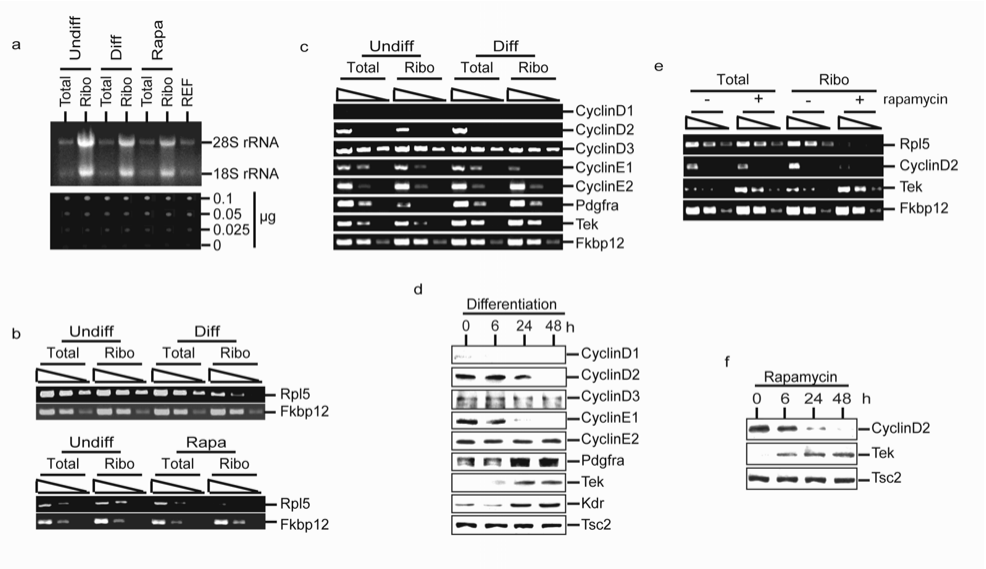
**Translational control during endothelial differentiation and rapamycin treatment**. a) Preparation of total RNA and ribosome-enriched RNA. RoSH2 cells, RoSH2 induced to differentiate by plating on matrigel for 48 hours and RoSH2 cells treated with 50 ηM rapamycin for 48 hours were harvested. Total and ribosome-enriched RNAs were quantitated by absorbance at 260 nm. 2 μg of RNA from each of the two cellular fractions were separated on a 1.0% agarose gel, stained with ethidium bromide and visualized under UV illumination. *Top panel *Representative RNA samples from total and ribosome-enriched RNAs prepared from undifferentiated (Undiff), differentiated (Diff) and rapamycin-treated (Rapa) RoSH2 cells are shown. *Bottom panel *For each sample, 1 μl of serially diluted RNA sample mixed with 1 μl of 0.5 ηg/μl ethidium bromide was visualized under UV illumination to verify RNA loading in each lane; RNA was isolated from total cellular extract (total) and ribosome-enriched subcellular fraction (ribo) prepared from RoSH2 cells before and 48 hours after induction of endothelial differentiation by plating matrigel-coated plates; b) Distribution of rpL5 mRNA in total and ribosome-enriched RNAs before and after differentiation. RT-PCR using oligo-dT-primed cDNA, and Rpl5 and Fkbp12 specific primers was performed on 10 fold serial dilution i.e. 1×, 10× and 100× of RNAs.; c) RT-PCR analysis of transcript abundance in RoSH2 cells before and after differentiation. RoSH2 cells were induced to undergo endothelial differentiation by plating on matrigel. Total and ribosome-enriched RNA were purified at 0 and 48 hours and analyzed by RT-PCR for transcript abundance of G1 cyclins and endothelial receptors; d) Western blot analysis. RoSH2 cells were induced to undergo endothelial differentiation by plating on matrigel and at 0, 6, 24 and 48 hours, cell lysates were prepared and assayed by western blot analysis. Tsc2 protein was used as an internal control for loading between lanes and between blots; e) Effect of rapamycin on Rpl5, cyclinD2 and Tek transcript abundance in total and ribosome-enriched RNA. Total and ribosome-enriched RNA were isolated from RoSH2 cells treated for 0 and 48 hours with 50 ηM rapamycin treatment and analyzed by RT-PCR; f) Western blot analysis. Cell lysates from rapamycin-treated RoSH2 cells at 0, 6, 24 and 48 hours were assayed by western blot analysis for cyclinD2 and Tek. Tsc2 protein was used as an internal control for loading between lanes and between blots.

Eif4ebp1 and Rps6kb1 function primarily to promote ribosomal recruitment of specific classes of mRNAs e.g. TOP-containing mRNAs [22]. We therefore examined if dephosphorylation of Eif4ebp1 and Rps6kb1 during differentiation or rapamycin-treatment resulted in reduced ribosomal recruitment of TOP containing mRNA or mRNAs associated with G1/S progression in cell cycle or endothelial differentiation. Total cellular extract and a cellular fraction that was enriched 3–4 fold in ribosomes were prepared from RoSH2 cells before and 48 hours after differentiation or rapamycin treatment. Enrichment of ribosomes was assessed by the relative increase in 18S and 28S ribosomal RNA (fig 6a). We observed that consistent with the involvement of Eif4ebp1 and Rps6kb1 during differentiation and rapamycin treatment, there was a concomitant displacement of a TOP containing mRNA, RNA ribosomal protein L5 (Rpl5) RNA from the ribosome-enriched fraction (fig 6b). There was no significant decrease in total Rpl5 RNA abundance. We next investigated if there were changes in ribosomal recruitment of mRNAs associated with G1/S progression in cell cycle or endothelial differentiation during differentiation and/or rapamycin treatment. As cyclinD1, a member of the G1 cyclins that regulate G1/S transition [23-25] has previously been shown to be regulated at the translational level through the PI3 K pathway [26,27], we examined the G1 cyclins consisting of cyclinD family of D1, D2 and D3 and cyclinE family of E1 and E2. RoSH2 cells did not express detectable levels of cyclinD1 transcript (fig 6c). Transcripts of the remaining four cyclins, D2, D3, E1 and E2 were detectable and their total transcript abundance remained unchanged during differentiation. However, transcript abundance of cyclinD2 and cycinE1 in ribosome-enriched RNA fraction was much reduced during differentiation and there was a corresponding decrease in their respective protein levels (fig 6d). We observed that in contrast to cyclinD2 and E1, the transcript abundance of several endothelial receptors e.g. Pdgfrα [28-30], Tek [31] and Kdr [32] was either increased or not reduced in the ribosome-enriched RNA fraction during differentiation (fig 6c; data not shown for Kdr) and their protein levels were increased.(fig 6d). Rapamycin treatment induced a similar response. Rpl5 and cyclinD2 transcript abundance were reduced in the ribosome-associated RNA fraction with similar reductions in their protein levels (fig 6e, f). Transcript abundance of an endothelial specific receptor, Tek was increased in the ribosome-associated RNA fraction and protein level (fig 6e, f). Consistent with rapamycin treatment, inhibiting PI3 K with either LY 294002or wortmannin also decreased cyclinD2 and increased Tek (fig 5a).

## Discussion

We have previously derived lineage-restricted stem cell lines from either embryos or ESCs as a strategy to circumvent some of the problems associated with the use of ESCs for replacement therapy, namely the risk of tumor formation and the efficiency of generating clinically useful cell types in large and homogenous quantity [3]. Towards this end, we have derived clonal RoSH cell lines from mouse embryos [4] and more recently, molecularly defined CD9^hi ^SSEA-1^- ^E-RoSH cell lines from mouse ESCs [3]. RoSH and E-RoSH cell lines are highly similar with a highly correlated genome-wide gene expression profile (r^2 ^= 0.93) [3]. They can be propagated in an undifferentiated state and can be induced to differentiate efficiently into endothelial cells *in vitro *and *in vivo*. To better manipulate these lines, we investigated the signaling process that regulates proliferation and differentiation of these lines.

We demonstrated that PI3 K/Akt/mTOR but not Raf/MEK/Erk signaling was the critical signaling pathway in the proliferation of undifferentiated RoSH cells. Inhibition of PI3 K/Akt/mTOR but not Raf/MEK/Erk signaling reduced cellular proliferation, increased accumulation of dividing cells in G1 phase, dephosphorylated Eif4ebp1 and Rps6kb1, abrogated ribosomal recruitment of specific classes of mRNAs e.g. TOP mRNAs and cyclinD2 mRNA and enhanced expression of endothelial receptors e.g. Kdr, Tek, Pdgfrα [33]. Consistent with these observations, PI3 K/Akt/mTOR but not Raf/MEK/Erk signaling was reduced when self-renewing RoSH2 cells were induced to differentiate. Differentiating RoSH2 cells manifested similar molecular characteristics of rapamycin-treated cells. The response was however generally more gradual than that observed in rapamycin-treated cells, possibly because inhibition of mTOR during differentiation was elicited through a cascade of cellular events unlike direct inhibition by rapamycin.

The major downstream targets of PI3 K/Akt/mTOR-mediated regulation are the major regulators of translational control, Rps6kb1 and Eif4ebp1 [5]. Both Rps6kb1 and Eif4ebp1 enhance translation of specific classes of mRNAs e.g TOP-containing mRNAs that include Rpl5 mRNA [22]. They also regulate G1/S progression in the cell cycle [9,11]. Here we demonstrated that in proliferating RoSH2 cells, Rps6kb1 and Eif4ebp1 were highly phosphorylated. CyclinD2 and Rpl5 transcripts were associated with ribosomes, and cyclinD2 was easily detectable at the protein level. When RoSH2 cells were induced to differentiate or treated with rapamycin, Rps6kb1 and Eif4ebp1 became dephosphorylated, and cyclinD2 transcripts were displaced from ribosome-enriched RNA fraction without a significant reduction in total RNA abundance and its protein product was significantly reduced. These observations suggest that cyclinD2 expression in proliferating RoSH/E-RoSH cells is maintained by active translation of its transcripts by PI3 K/Akt/mTOR signaling, and upon differentiation, its expression is downregulated through reduced PI3 K/Akt/mTOR signaling that resulted in reduced translation. In contrast, translation of endothelial specific Pdgfrα [28-30], Tek [31] and Kdr [32] transcripts were either increased or not reduced during differentiation, and their protein products were significantly increased, suggesting that translation of these transcripts is not regulated by PI3 K/Akt/mTOR signaling.

CyclinD2 is a G1 cyclin that promotes G1/S progression [23-25]. The association of translational regulation of cyclinD2 with PI3K/Akt/mTOR signaling provided a molecular basis for the downregulation of cell proliferation and inhibition of G1/S progression in cell cycle when PI3K/AKt/mTOR signaling was inhibited in differentiating RoSH2 cells or during treatments with inhibitors of PI3K/Akt/mTOR signaling. The increased protein level of endothelial specific receptors during downregulation of PI3K/Akt/mTOR signaling also provided a link between the inhibition of proliferation and the induction of differentiation. The rapid increase in protein levels of critical angiogenic or vasculogenic receptors such as Kdr and Tek [32] upon inhibition of proliferation in RoSH cells provided a molecular basis for the robust and efficient differentiation of RoSH/E-RoSH cells into endothelial cells. As previously reported, expression of these receptors on cell surface was robustly induced with a dramatic increase from <1% to >60% of the cells expressing either or both receptors within 60 hours of differentiation [3]. However, the role of PI3K/Akt/mTOR signaling in regulating the protein level of these endothelial receptors is clearly mechanistically different from that of cyclinD2.

Together, our observations in this study are consistent with a general view that mTOR-mediated translation regulation is important in regulating proliferation and differentiation of stem or progenitor cells including embryonic stem cells [34], and adult stem cells such as vascular smooth muscle progenitor cells [35], neural progenitor cells [36] and liver cancer cells [37]. More specifically, our observations are consistent with the view that a primary cellular response to mitogenic PI3K/Akt signaling pathway is the elicitation of differential translational regulation of specific mRNA subsets via coordinated activation and inactivation of the components of translational machinery and the general translational repressors [38]. This study also demonstrates that downregulation of PI3 K/mTOR signaling constitutes part of the molecular program necessary to elicit endothelial differentiation of endothelial progenitor cells. This is consistent with an earlier report that PI3 K is critical for survival, mitogenesis and migration but not for differentiation of endothelial cells [39], and with the observations that aberrations in PI3K pathway are implicated in many aspects of tumor angiogenesis [40]. More specifically, perturbations in rapamycin-sensitive mTOR activity generally result in abnormal vascularization (reviewed [41,42]). In particular, t*sc1+/- *or *tsc2*+/- mutant mice which are characterized by high level of mTOR-mediated phosphorylation of Eif4ebp1 and Rps6kb1, have hemangiomas with poorly developed vasculature that are prone to rupture [43-45]. Therefore, our observation that downregulation of PI3 K/mTOR signaling is a critical component in the proliferation and endothelial differentiation of RoSH2 endothelial progenitor cells provides a mechanistic basis for abnormal vascularization with functionally defective vasculature when this pathway is constitutively active in hamatomas [42]. We postulate that failure to downregulate PI3 K/mTOR signaling during endothelial differentiation prevents endothelial progenitor cells from exiting from cell cycle to properly initiate a differentiation program, resulting in proliferation of abnormally differentiated endothelial cells and formation of functionally defective vasculature.

## Conclusion

In conclusion, this study highlights the importance of translation regulation as a critical regulatory mechanism in the regulation of self-renewal and differentiation in stem cells. As demonstrated here, PI3 K/Akt/mTOR-mediated translation regulation was a dominant regulatory mechanism in the proliferation and endothelial differentiation of RoSH/E-RoSH cells. It was critical in maintaining a high level of proliferation-associated proteins such as cyclinD2 to drive cell cycle activity. Downregulation of this signaling either during differentiation or through use of small molecule inhibitors, inhibits translation of proliferation-associated cyclinD2 gene transcripts but not differentiation-associated markers.

## Methods

### Cell culture

The maintenance and differentiation of RoSH2 cells have been previously described [4]. To test the effects of PI3 K inhibitors, LY294002 and wortmannin on mTOR activity, cells were cultured for 24 h immediately after passaging and then deprived of serum for 20 h. Cells were then treated with or without 50 μM LY294002 (Sigma, St. Louis, MO) or 100 ηM wortmannin (Sigma, St. Louis, MO) for 15 min and then stimulated with 100 ηM insulin for 20 min. For other treatment, 80% confluent cultures were treated with 50 ηM rapamycin (Sigma, St. Louis, MO), 10 μM Ras inhibitor FTase inhibitor III (Calbiochen-Novabrochem Corp, La Jolla, CA) and 50 μM MEK inhibitor PD98059 (Calbiochen-Novabrochem Corp, La Jolla, CA).

### Proliferation assay

To assess cell cycle rate, 2 × 10^8 ^RoSH2 cells were pre-labeled with 2 ml of 10 μM CFDA (Molecular Probe, Eugene, Or) in saline at 37°C for 15 minutes, cultured in non-differentiating conditions for 24 hours and then replated at 1 × 10^5 ^cells per 3 cm dish under non-differentiating or differentiating conditions. For drug treatment, the cells were cultured in the presence of 50 ηM rapamycin, 50 μM LY294002, 10 μM Ras inhibitor FTase inhibitor III, or 50 μM MEK inhibitor PD98059. For each drug, the controls were treated with vehicle alone. At 0, 24 and 48 hours, three plates of cells were harvested, fixed in 2% paraformaldehyde, and analyzed on a FACStar^plus ^(Becton Dickinson; San Jose, CA). The number of cell cycles per 24 hours was calculated assuming that each halving of cellular fluorescence represented one cell division. Therefore, the number of cell cycles per 24 hours (n) was calculated as n = (lg F/F_n_)/lg 2 where F is initial average cellular fluorescence and F_n _is the average cellular fluorescence after 24 hours.

### Cell cycle analysis using BrdU

RoSH2 cells were plated on gelatin or matrigel for 24 hours before they were labeled for 16 hours with 10 μM BrdU (Sigma, St Louis). BrdU was then removed and fresh medium was added and were cultured for another 6 or 12 hours. For rapamycin treatment, cells were cultured on gelatin for 24 hours before pulse-labeling for 16 hours with 10 μM BrdU (Sigma, St Louis) after which 50 ηM rapamycin was added. Cells were fixed in ice cold 70% ethanol, treated with 2 N HCl/0.1% Triton-100× in PBS for 20 minutes at 37°C, and then incubated with FITC-conjugated anti-BrdU antibody (Pharmingen,) at 1:50 dilution, 30 minutes at room temperature. Cells were then counterstained with propidium iodide (10 μg/ml in PBS) in the presence of 1 mg/ml RNaseA for 30 minutes at room temperature and analyzed by flow cytometry.

### Preparation of total cellular and ribosome-enriched RNA

Total RNA were prepared using a modified method of Chomczynski and Sacchi as previously described [46,47]. Ribosomal RNA was prepared using a modified protocol for preparing polysomes [48]. Briefly, exponentially growing cells were harvested and resuspended at 10^8 ^cells per ml buffer (10 mM Tris-Cl, pH 7.6, 1 mM potassium acetate, 1.5 mM magnesium acetate, 2 mM dithiothreitol (DTT), 10% glycerol, 1 μg/ml leupeptin, 1 μg/ml pepstatin A, 100 μg/ml phenylmethylsulfonyl fluoride). Cells were homogenized in a pre-chilled Dounce homogenizer sitting in an ice-water bath and cell lysates were centrifuged at 9000 g for 10 minutes at 4°C. The resulting supernatant was layered over a cushion of 30% (w/v) sucrose in lysis buffer and centrifuged at 130,000 *g *for 2·5 hours at 2°C. The ribosomal RNA pellet was resuspended in acid-guanidinium thiocyanate buffer and RNA was purified using a CsCl gradient.

### RT-PCR

RT-PCR was performed as previously described [4]. Primer sets for amplification of the following genes and the expected amplified cDNA fragment size were a) FKBP12 5'-CAC GGG GAT GCT TGA AGA TGG-3' and 5'-GTC TAT ACA AAG GGT GGT GGG-3', 371 bp; b) PDGFRα 5'-CCA GTA GTT CCA CCT TCA TCA-3' and 5'-CAA GTA TCC CAG CTA TCC ACA-3', 275 bp; c) Tek 5'-CTG TTG GCG TTT CTG ATT ATG-3' and 5'-GGG TCT GTC TCT AGC ACT CTG-3', 482 bp; d) rpL5 5'-GCC TTC ACT TGC TAT CTG GAT-3' and 5'-CCT CTT CTT CTT CAC TTC TCT-3', 375 bp; e) Cyclin D1 5'-GTG AGG GAA GAG GTG AAG GTG-3' and 5'-GGT TTG GTT TTG CCC GTG GTG-3', 732 bp; f) cyclin D2 5'-GTA AGA TGC TTA CAG GAG AAC-3' and 5'-CCT CAC CCT CTT CCC TTA CAC-3', 585 bp; g) cyclin D3 5'-CGC AAT TGC AGC TTC T AG G TA-3' and 5'-CAT CCG CAG ACA TAG AGC AGG-3', 381 bp; h) cyclin E1 5'-CGC TGC TCT GCC TTC TTA CTG-3' and 5'-GTC CTC GCT GCT TCT GCT TTG-3', 326 bp; i) cyclin E2 5'-GAA ATC TAC GCT CCT AAA CTC-3' and 5'-GTG TTT TCC TGG TGG TTT TTC-3', 603 bp.

### Western blot analysis

Standard procedures were used [49]. Briefly, cells were lysed in RIPA buffer, centrifuged at 14,000 rpm for 15 minutes at 4°C and the supernatant was stored in aliquots at -70°C. 20 μg lysate was denatured, separated on 10 or 15% SDS-polyacrylamide gel and electro-blotted onto a nitrocellulose membrane. The membrane was incubated sequentially with a primary antibody, then either a HRP conjugated-secondary antibody or a biotinylated secondary antibody followed by neutravidin-HRP, and finally, a HRP enhanced chemiluminescent substrate, ECS (Pierce, Rockford, IL). Each membrane was probed sequentially with three primary antibodies. The membrane was stripped using 2% SDS, 100 mM β-mercaptoethanol and 50 mM Tris (pH 6.8) between exposure to each primary antibody. Primary antibodies were 1:200 dilution of rabbit anti-phosphoRps6kb1, Rps6kb1, Eif4ebp1, Tsc2, phosphoAkt1, cyclinD1, cyclinD2, cyclinD3 and cyclinE1, goat anti-cyclinE2 and anti-(4E-BP1) polyclonal antibodies (Santa Cruz Biotechnology, CA), and 1:500 dilution of anti-MAPK (p42/44) rabbit polyclonal antibody (Cell Signaling Technology, MA), and rat anti-Kdr and rabbit anti-Tek (PharMingen, MA). Secondary antibodies were HRP-conjugated goat anti-rabbit, rabbit anti-goat and rabbit anti-mouse. After exposure to three primary antibodies, ach membrane probed with rabbit anti-Tsc2 as an internal control for loading.

## List of abbreviations

ESCs – Embryonic stem cells, TOP – tract of pyrimidines, mTOR-mammalian target of rapamycin

## Competing interests

The author(s) declare that they have no competing interests.

## Authors' contributions

JQ did most of the experiments, QZ contributed to experiments listed in fig 1, 2, 3 and 4, RME contributed to the design and writing of the manuscript, BL contributed to the writing of the manuscript, S-KL lead and wrote the manuscript.

## References

[B1] Keller G (2005). Embryonic stem cell differentiation: emergence of a new era in biology and medicine. Genes Dev.

[B2] Wobus AM, Boheler KR (2005). Embryonic stem cells: prospects for developmental biology and cell therapy. Physiol Rev.

[B3] Lian Q, Yeo K, Que J, Tan E, Yu F, Yin Y, Salto-Tellez M, Oakley RM, Lim SK (2006). Establishing Clonal Cell Lines with Endothelial-Like Potential from CD9, SSEA-1 Cells in Embryonic Stem Cell-Derived Embryoid Bodies. PLoS ONE.

[B4] Yin Y, Que J, Teh M, Cao WP, El Oakley RM, Lim S-K (2004). Embryonic Cell Lines with Endothelial Potential: An In Vitro System for Studying Endothelial Differentiation. Arterioscler Thromb Vasc Biol.

[B5] Richardson CJ, Schalm SS, Blenis J (2004). PI3-kinase and TOR: PIKTORing cell growth. Semin Cell Dev Biol.

[B6] Chang F, Steelman LS, Shelton JG, Lee JT, Navolanic PM, Blalock WL, Franklin R, McCubrey JA (2003). Regulation of cell cycle progression and apoptosis by the Ras/Raf/MEK/ERK pathway (Review). Int J Oncol.

[B7] Haugland RP, Spence MT (1996). Probes for Live-Cell Function. Handbook of fluorescent probes and research chemicals.

[B8] Lawrence JC, Lin TA, McMahon LP, Choi KM (2004). Modulation of the protein kinase activity of mTOR. Curr Top Microbiol Immunol.

[B9] Fingar DC, Richardson CJ, Tee AR, Cheatham L, Tsou C, Blenis J (2004). mTOR controls cell cycle progression through its cell growth effectors S6K1 and 4E-BP1/eukaryotic translation initiation factor 4E. Mol Cell Biol.

[B10] Fingar DC, Blenis J (2004). Target of rapamycin (TOR): an integrator of nutrient and growth factor signals and coordinator of cell growth and cell cycle progression. Oncogene.

[B11] Schmelzle T, Hall MN (2000). TOR, a central controller of cell growth. Cell.

[B12] Inoki K, Li Y, Zhu T, Wu J, Guan KL (2002). TSC2 is phosphorylated and inhibited by Akt and suppresses mTOR signalling. Nat Cell Biol.

[B13] Wang L, Proud CG (2002). Ras/Erk Signaling Is Essential for Activation of Protein Synthesis by Gq Protein-Coupled Receptor Agonists in Adult Cardiomyocytes. Circ Res.

[B14] Nguyen KA, Santos SJ, Kreidel MK, Diaz AL, Rey R, Lawson MA (2004). Acute Regulation of Translation Initiation by Gonadotropin-Releasing Hormone in the Gonadotrope Cell Line L{beta}T2. Mol Endocrinol.

[B15] Lenormand P, McMahon M, Pouyssegur J (1996). Oncogenic Raf-1 activates p70 S6 kinase via a mitogen-activated protein kinase-independent pathway. J Biol Chem.

[B16] Yamakawa T, Tanaka S, Numaguchi K, Yamakawa Y, Motley ED, Ichihara S, Inagami T (2000). Involvement of Rho-kinase in angiotensin II-induced hypertrophy of rat vascular smooth muscle cells. Hypertension.

[B17] Kleijn M, Korthout MM, Voorma HO, Thomas AA (1996). Phosphorylation of the eIF4E-binding protein PHAS-I after exposure of PC12 cells to EGF and NGF. FEBS Lett.

[B18] Rocic P, Jo H, Lucchesi PA (2003). A role for PYK2 in ANG II-dependent regulation of the PHAS-1-eIF4E complex by multiple signaling cascades in vascular smooth muscle. Am J Physiol Cell Physiol.

[B19] Voisin L, Foisy S, Giasson E, Lambert C, Moreau P, Meloche S (2002). EGF receptor transactivation is obligatory for protein synthesis stimulation by G protein-coupled receptors. Am J Physiol Cell Physiol.

[B20] Kelleher RJ, Govindarajan A, Jung HY, Kang H, Tonegawa S (2004). Translational control by MAPK signaling in long-term synaptic plasticity and memory. Cell.

[B21] Shahbazian D, Roux PP, Mieulet V, Cohen MS, Raught B, Taunton J, Hershey JW, Blenis J, Pende M, Sonenberg N (2006). The mTOR/PI3K and MAPK pathways converge on eIF4B to control its phosphorylation and activity. Embo J.

[B22] Gingras AC, Raught B, Sonenberg N (2004). mTOR signaling to translation. Curr Top Microbiol Immunol.

[B23] Stacey DW (2003). Cyclin D1 serves as a cell cycle regulatory switch in actively proliferating cells. Curr Opin Cell Biol.

[B24] Ekholm SV, Reed SI (2000). Regulation of G(1) cyclin-dependent kinases in the mammalian cell cycle. Curr Opin Cell Biol.

[B25] Sherr CJ, Roberts JM (1999). CDK inhibitors: positive and negative regulators of G1-phase progression. Genes Dev.

[B26] Takuwa N, Fukui Y, Takuwa Y (1999). Cyclin D1 expression mediated by phosphatidylinositol 3-kinase through mTOR-p70(S6K)-independent signaling in growth factor-stimulated NIH 3T3 fibroblasts. Molecular And Cellular Biology.

[B27] Muise-Helmericks RC, Grimes HL, Bellacosa A, Malstrom SE, Tsichlis PN, Rosen N (1998). Cyclin D Expression Is Controlled Post-transcriptionally via a Phosphatidylinositol 3-Kinase/Akt-dependent Pathway. J Biol Chem.

[B28] Beitz JG, Kim IS, Calabresi P, Frackelton AR (1991). Human microvascular endothelial cells express receptors for platelet-derived growth factor. Proc Natl Acad Sci USA.

[B29] Beitz JG, Kim IS, Calabresi P, Frackelton AR (1992). Receptors for platelet-derived growth factor on microvascular endothelial cells. Exs.

[B30] Marx M, Perlmutter RA, Madri JA (1994). Modulation of platelet-derived growth factor receptor expression in microvascular endothelial cells during in vitro angiogenesis. J Clin Invest.

[B31] Sato TN, Tozawa Y, Deutsch U, Wolburg-Buchholz K, Fujiwara Y, Gendron-Maguire M, Gridley T, Wolburg H, Risau W, Qin Y (1995). Distinct roles of the receptor tyrosine kinases Tie-1 and Tie-2 in blood vessel formation. Nature.

[B32] Breier G, Damert A, Plate KH, Risau W (1997). Angiogenesis in embryos and ischemic diseases. Thromb Haemost.

[B33] Ostman A (2004). PDGF receptors-mediators of autocrine tumor growth and regulators of tumor vasculature and stroma. Cytokine Growth Factor Rev.

[B34] Takahashi K, Murakami M, Yamanaka S (2005). Role of the phosphoinositide 3-kinase pathway in mouse embryonic stem (ES) cells. Biochem Soc Trans.

[B35] Martin KA, Rzucidlo EM, Merenick BL, Fingar DC, Brown DJ, Wagner RJ, Powell RJ (2004). The mTOR/p70 S6K1 pathway regulates vascular smooth muscle cell differentiation. Am J Physiol Cell Physiol.

[B36] Rajan P, Panchision DM, Newell LF, McKay RD (2003). BMPs signal alternately through a SMAD or FRAP-STAT pathway to regulate fate choice in CNS stem cells. J Cell Biol.

[B37] Parent R, Kolippakkam D, Booth G, Beretta L (2007). Mammalian Target of Rapamycin Activation Impairs Hepatocytic Differentiation and Targets Genes Moderating Lipid Homeostasis and Hepatocellular Growth. Cancer Res.

[B38] Evdokimova V, Ovchinnikov LP, Sorensen PH (2006). Y-box binding protein 1: providing a new angle on translational regulation. Cell Cycle.

[B39] Qi JH, Matsumoto T, Huang K, Olausson K, Christofferson R, Claesson-Welsh L (1999). Phosphoinositide 3 kinase is critical for survival, mitogenesis and migration but not for differentiation of endothelial cells. Angiogenesis.

[B40] Brader S, Eccles SA (2004). Phosphoinositide 3-kinase signalling pathways in tumor progression, invasion and angiogenesis. Tumori.

[B41] Tee AR, Blenis J (2005). mTOR, translational control and human disease. Semin Cell Dev Biol.

[B42] Inoki K, Corradetti MN, Guan KL (2005). Dysregulation of the TSC-mTOR pathway in human disease. Nat Genet.

[B43] Kobayashi T, Minowa O, Kuno J, Mitani H, Hino O, Noda T (1999). Renal carcinogenesis, hepatic hemangiomatosis, and embryonic lethality caused by a germ-line Tsc2 mutation in mice. Cancer Res.

[B44] Kwiatkowski DJ, Zhang H, Bandura JL, Heiberger KM, Glogauer M, el-Hashemite N, Onda H (2002). A mouse model of TSC1 reveals sex-dependent lethality from liver hemangiomas, and up-regulation of p70S6 kinase activity in Tsc1 null cells. Hum Mol Genet.

[B45] El-Hashemite N, Walker V, Zhang H, Kwiatkowski DJ (2003). Loss of Tsc1 or Tsc2 Induces Vascular Endothelial Growth Factor Production through Mammalian Target of Rapamycin. Cancer Res.

[B46] bin Ali A, Zhang Q, Lim YK, Fang D, Retnam L, Lim SK (2003). Expression of major HDL-associated antioxidant PON-1 is gender dependent and regulated during inflammation. Free Radic Biol Med.

[B47] Chomczynski P, Sacchi N (1987). Single-step method of RNA isolation by acid guanidinium thiocyanate-phenol-chloroform extraction. Analytical Biochemistry.

[B48] Caruccio N, Ross J (1994). Purification of a human polyribosome-associated 3' to 5' exoribonuclease. J Biol Chem.

[B49] Yin Y, Lim YK, Salto-Tellez M, Ng SC, Lin CS, Lim SK (2002). AFP(+), ESC-Derived Cells Engraft and Differentiate into Hepatocytes in Vivo. Stem Cells.

